# Scorpion Peptides: Potential Use for New Drug Development

**DOI:** 10.1155/2013/958797

**Published:** 2013-06-15

**Authors:** BenNasr Hmed, Hammami Turky Serria, Zeghal Khaled Mounir

**Affiliations:** Laboratory of Pharmacology, Medicine Faculty of Sfax, Street of Majida Boulila, 3029 Sfax, Tunisia

## Abstract

Several peptides contained in scorpion fluids showed diverse array of biological activities with high specificities to their targeted sites. Many investigations outlined their potent effects against microbes and showed their potential to modulate various biological mechanisms that are involved in immune, nervous, cardiovascular, and neoplastic diseases. Because of their important structural and functional diversity, it is projected that scorpion-derived peptides could be used to develop new specific drugs. This review summarizes relevant findings improving their use as valuable tools for new drugs development.

## 1. Introduction

Recently, natural products are sought with great concern for their contributions in basic researches for new drugs discovery [1]. In 2004, Clardy and Walsh reappraised that 23% of newly generated drugs with approvals from the US Food and Drug Administration (FDA) relied on naturally occurring substances [2]. In this issue, many toxins with high specificity to target cellular elements are sought to be used against many diseases [3]. To optimize their contributions as medicinal tools, outlining these substances pharmacognosy is a necessity [4]. Several decades ago, scorpion originating-peptides have been isolated and purified and gained much attention for their attributes in specifically targeting various ions channels and cell membrane components. These arachnid secretions amount to 800 isolated and characterized native and recombinant biologically active polypeptides, and most of them are endowed with strongly stabilized structure, but their total number was estimated to reach 100 000 different ones [5]. Eventually, they might offer a promising scaffold for new drugs development [6–11]. Thus, reviewing this emergent quality of scorpion derivatives constitutes the goal of this paper.

## 2. Vernacular and Toxicology

More than 1500 different known scorpion species have been described, around the world [12]. Classically, these arachnids are divided into two different groups, alongside with their geographical distribution: the old and the new world scorpions. The formers are essentially distributed in Africa, Asia, and Southern America. They are gathered into one family, the Buthidae clustering species with triangular-shaped sternum. However, new world scorpions, with a pentagonal-shaped sternum, are widely dispersed in Europe, Asia, and America. This suborder, namely, the Chactidae, comprises six different families (Scorpionidae, Diplocentridae, Chactidae, Vaejovidae, Bothriuridae, and Chaerilidae). It is worth mentioning that most dangerous species are comprised in the Buthidae family [13–15]. Unlike animals from the Buthidae family, which secrete potent mammalian-neurotoxic compounds, the Chactidae scorpions produce cells lytic and cytotoxic substances [16–18]. The occurrence of scorpion stings in humans was estimated to one million persons with a fatality risk of about 3‰, annually [19]. This hazardous accident is manifested by a broad range of symptoms varying from localized pain to harmful cardio-respiratory arrest and coma [19]. Important experimental and clinical findings outlined that the observed pathological signs, essentially, arose from the direct or indirect activities of multiple neurotoxins contained in the venom that disturbs cell membrane potentials, especially those of excitable cells (neurons, myocytes, and cardiomyocytes) [20, 21]. Expectedly, the newworld scorpion' envenoming induces much cellular lyses, necrosis, and ulcers such as those caused by *Hemiscorpius lepturus* [18]. To ameliorate the wound healing of scorpionism, patients have been categorized into different classes based on the root of systemic alterations they manifested. According to Goyffon and Chippaux, the severity of scorpion envenomation may be conveniently ranked into four grades: benign, moderate, severe, and worst. The exclusive medical care is based on antivenom sera injection. But when the antivenom administration is overdue, it seemed to lack efficacy, and cardiovascular and respiratory functions reanimations are recruited especially in critical cases [22].

## 3. Scorpion Venoms

Scorpion venom is a mixture of polypeptides, nucleotides, lipids, mucoproteins, biogenic amines, and other unknown substances. The amounts of the produced compounds are variable and might rely on the animal specimen and the number of stings (and eventually of extractions). Noticeably, scorpion derivatives with enzymatic activities are less represented [23]. The total peptide contained in the venom did not exceed 5% of its dried weight [14]. This fraction contains polypeptides that are typically divided into different groups in relation to their structures, targetedsites, pharmacological relevance, and toxicities for mammals and/or insects and crustaceans [13, 24]. A broad range of bioactive peptides are already purified and characterized from scorpion venoms, with a total number estimated to approach 100 000 different ones, among them only 1% is mostly known [5]. These peptides clustering is still debated. However, regular families are yet under consideration. In conjunction with their targeted ion channels, four different families are considered: peptides modulating sodium, potassium, chloride, or calcium-gated channels. These neurotoxins are probably originating from a common ancestral native peptide [25] (for more details see [23]). Other compounds consisting almost of short peptides exhibited antimicrobial (AMPs) and bradykinin potentiating (BPPs) activities. These later are characterized by free cysteine residues [26]. Fewer scorpion peptidic derivatives have shown enzymatic activities similar to phospholipase A2 [27, 28], lysozyme C [29], and hyaluronidase [30] (Table 1).

Peptides affecting ion traffic throughout the cell membrane (neurotoxins) are by and large studied. Dealing with their pharmacological relevance and toxicity, they were subdivided into various subfamilies. Their higher specificities and affinities to various components of many gated ion channels warrant their use as pharmacological tools to probe ions gated pores functions and electrophysiology, thus improving our knowledge about the cellular functionality. Scorpion neurotoxins have a tightly stabilized tridimensional-shaped backbone by three or four disulfide bridges. This property lowered their in-vivo degradation; and expectedly increased their effective binding-time and action [20, 36]. The advent of new tools for molecular engineering intensively prompted the formulation of various peptides chimera, and many recombinant scorpion peptides had been generated.

Even as they have divergent targeted sites, scorpion bioactive derivatives exhibited allosteric interference activities, in a manner to amplify their biological effects [37]. Mainly, neurotoxins bind to surface membrane receptors (sites), but many peptides penetrate the cell membrane and activate components at its cytosolic interface; for example, maurocalcine and imperatoxin A penetrate the membrane bilayers and activate the intracellular ryanodine receptor to provoke the intracellular sequestrated calcium release [31–35]. 

The colossal structural and pharmacological diversity of scorpions peptides encourage various approaches for their use in new drugs-development. Herein, we attempted to gather some featured clinical usefulness of these peptides.

## 4. Antimicrobial Activities

Antimicrobial peptides (AMPs) have been isolated from a wide variety of animals and plants. They are cationic and amphipathic peptides, mostly within 50 amino acid residues, and were gathered into different groups. Their modus operandi remains discussable. Some AMPs function by disrupting the cell membrane, while others use different mechanism of action. Three proposed mechanistic models have been proposed to explain the cell membrane disruption: the “barrel stave,” “micellar aggregate,” and “carpet” ones, (reviewed in [38]) (Table 2).

Several scorpion-derived AMPs can be set as *α*-helical free-cysteine peptides which alter the cellular membrane structure [61]. Androctonin, a 25-residue disulphide-bridged peptide originating from *Androctonus australis* venom, exhibited potent antigrowth effect on both gram-positive and -negative bacteria. This antibiotic activity is accomplished by membrane disruption and leakage of pathogenic cells. Interestingly, in ex vivo experiments, this peptide did not affect mammalian erythrocytes, a foreseeable attribute for clinical application. Furthermore, androctonin induced a significant decrease in oxygen consumption and ATP generation and thereby will abolish the pathogen energetic machinery [50, 51].

Modulating the calcium intracellular signaling is another mechanism to inhibit bacterial growth. Accordingly, parabutoporin and opistoporins, respectively, isolated from *Parabuthus schlechteri* and *Opistophthalmus carinatus* scorpions, interact with coupled G proteins and consequently modulate intracellular calcium signaling and exert their antibacterial effects [45, 62]. These findings point toward two different targeted sites for these peptides: an intracytoplasmic site implying an interaction with intracellular components such a DNA, RNA, and enzymes; and constrained action on outer membrane sites [38].

In addition to direct membrane disruption, the external effect of scorpion toxins could be mediated through their binding to definite gated ions channels, similar to calcium-dependant potassium pores involved in microbes biology [63]. The repertoire of scorpion peptides contains a lot of these channel blockers that could be used as antibiotics. 

Other scorpion-derived AMPs repressed microbial growth through their phospholipase activity [27, 28]. Guillaume and her colleagues [27] reported that a three disulfide-bridged peptide, the Imperatoxin I (a 75 amino acid stranded peptide from the venom of *Pandinus imperator* scorpion), exhibited a Phospholipase A2 activity that inhibits the intra-erythrocytic development of *Plasmodium falciparum* which causes the most severe forms of human malaria. It is likely that this peptide interacts with the infected erythrocytes membrane lipids [27] or plasma-free fatty acids and liberates lipid products (peroxides) [64] leading to infection demise. The Imperatoxin I completely inhibited both fecundation and ookinete formation within in vitro micromolar concentrations [53]. A pioneer assay to combat malaria at its biological cycle was advanced by Possani and her collaborators (2002). They had produced a resistant transgenic vector to malaria transfecting genes encoding the imperatoxin that abolished the parasite biological cycle [55].

The poor selectivity of scorpions AMPs to pathogens is a major challenge for their clinical application. With the allowance of molecular engineering tools, Lee et al. had synthesized a pool of analogous for IsCT, which is a 13 amino acids-residue purified from* Opisthacanthus madagascariensis,* exhibiting high selectivity to bacterial membranes and keeping soft mammalian cells. They found that substitution of the Pro for Gly^8^ and Lys for Glu^7^ and Ser^11^ improves the cationic charge of the native IsCT molecule and allows it to efficiently bind to negatively charged phospholipids of bacteria [49]. Numerous AMPs are continually isolated from scorpion transcriptomes [39, 44, 46, 65–69] that would alleviate pathogens resistance to conventional drugs.

## 5. Homeostasis and Rheology

Among toxicological patterns of scorpion envenoming is the alteration of the hemodynamic and cardiovascular functions which are mediated through either direct or indirect effects of neurotoxins. Interestingly, several components of the venom are sought as potential remedies for many blood and rheology injuries. The mostly spectacular is the use of scorpion venom in Chinese ethnopharmacy to improve blood rheology and homeostasis [74].

A common challenge in cardiovascular and thrombosis disorders is to surpass the drugs efficiencies limitation by patients resistance and unfavorable side outcomes [75]. Since coagulation and/or its regulator factors perturbations have been observed after scorpion stings [76], it is proposed that the whole scorpion venom or its components could intervene in controlling platelet aggregation. The SVAP, an active peptide isolated from the scorpion *Buthus martensii *Karsch, improves the mesenteric microcirculation and blood rheology by decreasing the blood viscosity [74]. Later, it was proved that it inhibits the thrombosis formation parameters (inhibition of platelet aggregation and prolongation of the thrombosis occlusion time) in ex vivo and in vitro experiments, in a dose-dependent manner [77]. It was concluded that SVAP increases the generation of prostaglandin I2 which is an important thrombosis regulator [77]. Further clinical and experimental findings accounted for the perturbation of the balance between pro- and anti-inflammatory cytokines and prostanoids production, after scorpion envenomation [78–82], implying that such endogenous compounds will mandate the scorpion venom antithrombotic effect [75]. 

To some extent, thrombosis control could be operated through direct inhibition/activation of the platelet membrane permeation to potassium ions through voltage-operated and Ca^2+^-activated channels [83]. Such formulation instigated Wolfs et al. to investigate the effect of charybdotoxin, a scorpion peptide obstructing the calcium-activated potassium channels with intermediate conductance, on platelet function [84]. Charybdotoxin acts by decreasing the prothrombinase activity as well as the exposition of phosphatidylserine which is an outer surface membrane aggregating factor [85]. Furthermore, Borges et al. showed that the whole venom of the Brazilian scorpion *Tityus serrulatus* modulates the blood clot formations via platelet-activating factor receptor (PAFR) function alteration [86].

Another alternate use of scorpion peptides in cardiovascular therapy is to regulate blood vasomotion via the renin-angiotensin system inhibition. For that reason, Hodgson and Isbister have reviewed the potential application of a variety of venomous animal extracts to cardiovascular drug discovery. They mentioned that a pool of bradykinin potentiating peptides (BPPs), extracted from a variety of snakes and scorpions, inhibit the downbreaking of the endogenous bradykinin and the synthesis of the angiotensin II (vasoconstrictor). Such effects lead to the reduction of the systemic blood pressure [10]. Else more, hypotensins, extracted from the *Tityus serrulatus* scorpion venom, induced hypotension but without intervening in the angiotensin converting enzymes activity. Their mechanism of action is thought to be mediated via nitric oxide releasing and offers a second stratagem for the disease wound healing [87].

## 6. Immune Diseases

In 1980, Brahmi and Cooper reported that the native *Androctonus australis* hemolymph and its partial fraction 1 (eluted by chromatography in G-200 Sephadex column at a maximum of 280–340 nm, with 0.01 molarity and an elution pH of 8.05) stimulated the human, rabbit, and mouse lymphocytes mitogenesis in in vitro studies. Also, they did show that it triggered the lymphocytes erythrocytes agglutination which is reversed by sugar derivatives [88]. Twenty years later, approval of such immune cells function enhancement by *Tityus serrulatus* was provided. This leukocytosis was assumed to result in great part from the significant release of neutrophils from the bone marrow to blood vessels bed. The mechanism of this mobilization did involve the platelet-activating factor (PAF) receptor signaling [86]. Since it appears that scorpion venom is capable of modulating lymphoid cell lines proliferation and/or activities. More recently, five different fractions from the above mentioned venom (*T. serrulatus* venom) obtained by gel filtration chromatography were assayed for their potential to modulate immune peritoneal macrophages secretions. These later contributed to a differential modulation of macrophages function and could probably interact with each other in a synergistic manner [89]. Among them an isolated *γ*-Ts toxin accomplishes its immune regulatory effect through pro- and anti-inflammatory factors release [82].

In view of the fact that an important role was attributed for shaker potassium channels in the regulation of immune cells, Beeton and her colleagues had proved that a peptide (ShK (L5)) isolated from the sea anemone (*Stichodactyla helianthus*) suppresses the proliferation of human and rat T_EM_ cells and inhibits the IL-2 production at very low doses. Such effects are subsequent to the voltage-sensitive potassium channels (Kv1.3) inhibition. This peptide is able to prevent the experimentally induced autoimmune encephalomyelitis and suppress the hypersensitivity in rats [90]. In a similar manner, charybdotoxin isolated from *Leiurus quinquestriatus* venom blocked the voltage-gated potassium channels in human and murine T lymphocytes and suppressed their proliferation [91]. The inhibition of both voltage-sensitive (Kv1.3) and Ca^2+^-activated (with intermediate conductance) potassium channels modulates the membrane potential of the T cell, in a manner to sustain an elevated level of intracellular-free calcium which is essential for the first steps of their activation. Since that, more than 12 scorpion toxins which may block potassium channels in T cells with K_*d*_ ranging from picomolar to micromolar values, were suggested to covnteract immune diseases progression [92].

Moreover, parabutoporin an isolated AMP from *Parabuthus schlechteri* scorpion venom can activate the exocytosis and chemotaxis and inhibit superoxide production in human polymorphonuclear granulocytes, at submicromolar concentrations [93]. Chemotaxis was also observed using *Tityus serrulatus* scorpion venom that activates the complement system which takes part of unspecific immune sentinel [94]. The mechanism of such effect is mediated by the Rac receptor, involved in Chemotaxis and exocytosis stimulation, probably through the activation of G proteins. The *α*-helical amphipathic sheet of the scorpion AMP permits its insertion into the membrane to trigger the G proteins activation and thus prevents the NADPH oxidase complex function [95]. NADPH oxidase inhibition could also be triggered via the parabutoporin-p4^7phox^ interaction leading to PKC pathway stimulation [96]. More recently, Remijsen et al. deduced that the main activity is a consequence of an indirect stimulation of Akt following lipid rafting [97, 98].

Recent investigations showed that many AMPs, originating from scorpions, and their artificially generated analogues exhibit potent antiviral activities against measles, SARS-Cov, H5N1 [70], hepatitis B [71] and C [72], and HIV-1 [40, 73, 99] viruses (Table 3). Chiefly, these peptides operate through a direct disruption of the viral envelope and consequently decrease the infectivity of the pathogens. Exceptionally, peptides directed against HIV-1 adopted another pathway. In fact, a structurally modified scyllatoxin, a derived scorpion peptide, efficiently inhibited the binding of gp120 to CD4 in a competitive manner and thus suppressed the infection of CD4+ lymphocytes by human immunodeficiency viruses [100, 101]. Similarly, the Kn2-7 and mucroporin-S1 scorpion-derived peptides have been shown to exert potent anti-HIV actions via the inhibition of chemokines receptors CCR5- and CXCR4-mediated activities and replication of the viruses [73]. These findings might improve the anti-AIDS therapy.

## 7. Neurological Diseases

Nervous system activities are chiefly governed by gated ion channel pores frameworks. These later modulate ion traffic through the cellular membrane and regulate the firing and propagation of action potentials which are responsible for signal transmission. Any aberrant pores components expression and/or function would result neurological diseases. Because of their incontestable high specificity and affinity to various components of ions-gated channels, scorpion neurotoxins are featured as a potential candidate for neurological drug development [104]. 

In this topic, the “magic” *Buthus martensii *Karschscorpion is widely used in Chinese ethnomedicine to treat some neurological diseases such as apoplexy, epilepsy, and cerebral palsy [116]. Recently, evidence upon the antinociceptive effect of some of its constituents was provided [104].

The mandatory role of voltage-sensitive sodium channels in pain physiopathology and its treatment inspired the use of these channels blockers as remedies (reviewed by Dib-Hajj et al. [117]). In this sense, it had been shown that Bmk AS isolated from *Buthus martensii *Karsch induced a significant antinociceptive effect via the inhibition of the voltage tetrodotoxin-sensitive sodium current in sensory nerves [105, 106]. Anti-nociception was also induced by alpha-anatoxin Amm VIII, a weak modulator of Na(v)1.2 channel, and the depressant insect-selective beta-toxin LqqIT2, in a mechanistic scheme involving opioid receptors activities [118].

Presumably, the reinstatement of the hyperexcitability of sensory nerves is a meeting point for wound healing various neurological diseases like allodynia and hyperalgesia. Since that, toxins acting on ion permeability of nerves membranes are sought to be in the top focus for neurological drug discovery.

In multiple sclerosis, which is an inflammatory disease of the central nervous system, there is destruction of myelin sheath of the nerves which is associated with a relative axonal sparing. Such structural disarrangement results in conduction deficits. To prevent neurological signs and symptoms evoked in sclerosis, potassium effluxes blockers are recruited. Multiple potassium channels blockers with different affinities and specificities are extracted and purified from scorpion venoms and might improve treatment of the symptomatic table developed by multiple sclerosis patients [103].

Earlier reports showed that scorpion venoms interact with neurotransmitter receptors such as dopaminergic [119, 120] and G protein coupled like adrenergic and cholinergic receptors [121]. They probably did block them or influence their release [122]. More recently, Sudandiradoss and her colleagues proved the docking interaction of ten different scorpion neurotoxins with the D2 dopamine receptor and their antagonizing effects [102]. These neurotoxin interactions will perhaps prompt dopamine receptors targeting for treating schizophrenia and Parkinson's disease [123].

## 8. Cancer

**Figure 1:**
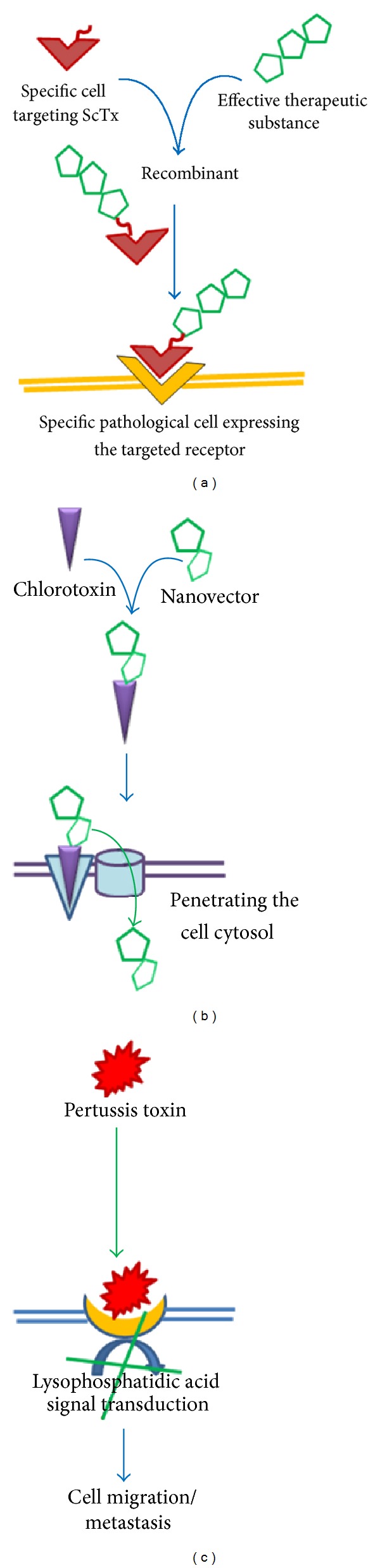
Schematic proposed models for scorpion toxins use in cancer and metastasis therapy. Scorpion toxins are dotted with high specificity to targeted specific cells receptors enabling them to be potent candidate for drugs carrier. (a) Radioactive iodine delivery into brain gliomas using chlorotoxin as a guider to target cancerous cells. (b) The chlorotoxin triggers gene transfection for cancer cells therapy. (c) Direct effect of pertussis toxin in abolishing metastasis. It did inhibit migration (evasion) through blocking the transduction signal of the lysophosphatidic acid pathway.

Cytotoxic compounds that kill cells or repress their growth are a required attribute for cancer and malignant diseases chemotherapy [124]. Unfortunately, the poor specificity of chemotherapeutic substances to render them less efficacious [125]. The ability of natural toxins to bind specifically to various cellular domains upholds new hope for anticancer drug development, like bombesin and bombesin-like toxins which have been used as drug motifs carriers with high specificity against tumoral cells [126–130]. This property is an attribute of chlorotoxin, a peptide extracted from the venom of *Leiurus quinquestriatus hebraeus*, which specifically binds to chloride-gated channels [107, 131–133] that are firmly involved in cancer cells mobility mechanism [134]; and impairs the in vitro glioma invasion [108–110]. Veiseh and her colleagues [135–137] examined the utility of the chlorotoxin as a nanovector carrier for gene transfection into both C6 glioma and DAOY medulloblastoma cells and improved its effectiveness as a good tool for clinical use (Figure 1) [136, 137]. Now, the chlorotoxin-like repertoire comprises other peptides such as the recombinant BmkTa, isolated from are *Buthus martensii *Karsch, that abolishes tumoral cells growth, but not normal ones [111]. 

The list of scorpion peptides exhibiting anti-proliferative and cytotoxic effects on tumor and malignant cells is expanding, and new substances are being continually added [126, 138–142].

Various types of potassium current, like those of the human ether-a-go-go-related potassium channels [143] and Kv11.1 [144] and Kir4.2 channels [145] are involved in metastasis and tumor growth [112, 146]. For example, outward current of potassium through the voltage-gated pore rectifier (Kir4.2) enhances the integrin-mediated cellular migration and dissemination [145]. Since that, hampering these gated pores using peptides, such as AmmTx3 (*Androctonus mauretanicus mauretanicus* toxin 3), BmTx3 (*Mesobuthus martensii* toxin 3), Bekm-1 (*Mesobuthus eupeus* toxin 1), and many other potassium scorpion toxins, could convey a promising field in counteracting the evolution of metastasis [112]. Recently, it was approved that voltage-gated sodium channels are functionally overexpressed in various types of human cancers, and correlate with metastatic progression [147, 148]. Seemingly, they are involved in the metastatic process through their interactions with different kinds of cytoplasmic proteins and enzymes, such as the adenylate cyclase and phosphokinases which mediate signals transduction regulating cellular motility [148]. Targeting these channels by specific scorpion-derived blockers could potentially suppress cancer cells functionalities. Phase I trials have been advanced using Pertussis toxin as “adjuvant” before radical cystectomy in bladder carcinoma and had promising effects against micrometastasis and neoplastic regeneration (Figure 1) [149, 150].

Hyaluronidases, endogenous enzymes (endoglycosidases), are scattered with a battery of diverse effects regulating cells activities and growth. They are involved in cell cycle progression, aging processes, and apoptosis. At some extent, they are also used for various objectives in cancer treatments. For example, they did facilitate drugs penetration and biodistribution [151]. Such enzyme, called BmHYA1, had been extracted and purified from the venom of the Chinese scorpion *Buthus martensii*. The BmHYA1 impairs the extracellular matrix receptor III (CD44) surface marker [30] that is overexpressed in cancer cells and promotes the matrix adhesion [152].

Wei-Dong and colleagues, a scientific group from the Institute of Basic Medicine (Shandong University (health sciences)), China, spare great effort to explore the usefulness of scorpion polypeptides in combating cancer diseases. They provided evidence for the potent inhibition of tumor development and metastasis processes in prostate, W256 sarcocarcinoma, liver, pancreas, DU-145, H-22 hepatic, S-180 sarcoma, BT474 breast, and SKOV-3 ovarian cancerous cell lines, in in vitro and in vivo transplantation mice models, by scorpion derivatives. The mode of action of the used polypeptides is still discussed and seems to trail different pathways.

In 2010, Xu and her coauthors had mentioned that a PESV fraction from a scorpion venom exhibited potential antitumoral activity against Lewis lung cancer cells which were inoculated in mice. PESV seems to act through the immune sentinel stimulation by decreasing VEGF, TGF-beta1, and IL-10 expressions in tumor environment and enhancing the overexpression of costimulatory molecules CD80 and CD86 in dendritic cells infiltrating the tumor [113]. Such findings will probably prompt the breaking down of immune tolerance to cancerous cells and improve cellular therapeutic strategies. Other peptides enhance the expression of P21 and caspases-3 proteins, members of the programmed cell death [141], or inhibit the expression or the activity of matrix metalloproteinase-2 and alter the *β*-catenin localization [126]. 

Relative to the protease inhibitor-based treatments against invasive cancer cells [153], a monomeric glycoprotein with 120 KDa, purified from *Heterometrus bengalensis*, could contribute in this anticancer treatment [154]. Similarly, BmAP1 will do so, the analogue of serine protease inhibitor which was extracted from *B. martensii Karsch* venom [155]. 

## 9. Osteoporosis

*Heterometrus bengalensis* scorpion venom exerts antiosteoporotic effects on ovariectomized female albino rats treated with methylprednisolone. The effect of the scorpion venom is thought to be directed on osteoclasts. It appears that it did increase the bone mineral deposit in conjunction with the modulation of involved regulatory factors (hormones, enzymes, and cytokines) [114]. Earlier report of Valverde et al. (2004) showed that blocking voltage-sensing potassium channels, using Kaliotoxin which is a Kv1.3 scorpion toxin blocker, reduces the inflammatory bone resorption. These activities will probably lead to the amelioration of bone resorption treatment [115].

## 10. Concluding Remarks

**Figure 2:**
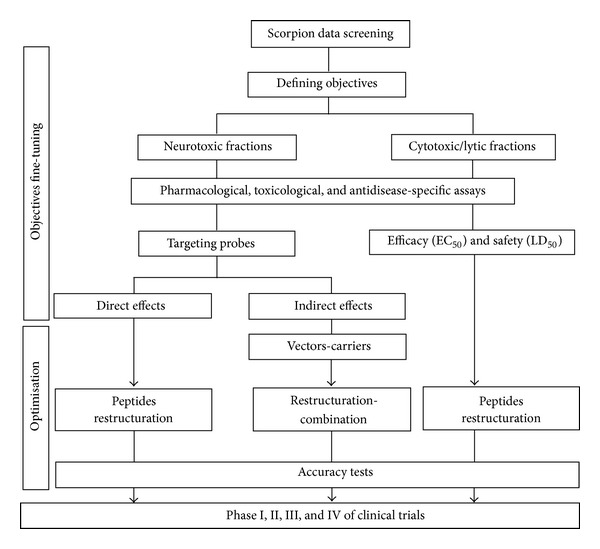
Schematic model representing the process in need to distillate scorpion derivatives with potential clinical use.

Scorpion venom and hemolymph offer an extendable inventory of polypeptides with diverse array of bioactivities and high specificity to definite elements of the cell. These polypeptides are often of low molecular weights and compactly stabilized with disulphide bridges. These assets put ahead their potential use as candidate for new drugs development (Table 4), but further investigations are required to commence their clinical applications. Nonetheless, phase I trials have been advanced for some peptides. One of the major limitations for scorpion substances to be clinically applied is their toxicity and nonselective patterns for the adducted or pathogenic cells. To disclose the matter, proteins and genes engineering prompted new techniques and tools to calibrate toxicity and restrict targeting components and cells by the peptide, simply by slight modifications in their primary sequence. Perhaps in the next few years, some newly isolated native peptides will respond in a fitted way to their pharmaceutical use. Besides, it is important to orient “scorpiology” research toward an accurate plot of characterization and testing bioactivities. The connotation of such plan is to predict the “desirable” scorpion and the substance in need. For example, to isolate an anticancer cytotoxic peptide, it would be prejudicially to use animals from the Chactidae family because of the known lytic and cytotoxic effects of their venoms. Once the venom (or its derivative) fits the sought clinical objectives, through the 1st series of bioassays, serial biochemical, pharmacological, and toxicological experiments will isolate and characterize the substance in need and improve its efficacy and safety. As a consequence, objectives will be fine-tuned to make decision for continuation. To optimize the pharmaceutical pattern of the extract, further biochemical and genetic intervention will be required, before running clinical trials. The art of this plan is the distillation of the tremendous researches in scorpiology toward clinical applications (Figure 2). Consistently, huge and sparse plethora of scorpion venoms and hemolymph analysis is ongoing but remains focusing on the isolation, purification, and characterization of sequences. We contemplate for additional experimental simple tests, like cytotoxicity against tumoral cells or pathogens during the characterization steps, which should be carried out to accurately redirect these peptides for basic researches for drugs development.

## Figures and Tables

**Table 1:** List of various families of polypeptides isolated from scorpion venom and hemolymph.

Family	Subfamilies	Targeted site
Peptides acting on Na^+^ channels		

	*α*-Mammals (classic)	
*α*-NaTxs [23]	*α*-Like	Site 3
	*α*-Insect	
	*β*-Mammals	
*β*-NaTxs [23]	*β*-Insect (depressant)	Site 4
	*β*-Insect (contracturant)	

Peptides acting on K^+^ channels		

*α*-KTXs [23]	In conjunction with the appropriate targeted site.	Kv1.x channels
		KCa1.1
		KCa2.x
		KCa3.1
		Kv11.x
*β*-KTXs [23]	Nd	Nd
*γ*-KTXs [23]	Nd	Kv11.x
*κ*-hefutoxin [23]	Nd	Kv1.x

Peptides acting on Cl^−^ channels		

Nd [23]	Nd	Ca^2+^-activated Cl^−^ channels (CFTR)

Peptides acting on Ca^2+^ channels		

Nd [31–35]	Nd	Ryanodine receptor

Peptides with free cysteine residues		

BPPs [10]	Nd	Nd
AMPs [26]	Nd	Membrane phospholipids

Peptides with enzymatic activities		

PLAs2 [27]	Nd	Nd
Hyaluronidases [30]	Nd	Nd

**Table 2:** List of antibacterial peptides isolated from scorpions.

Scorpion	Fraction	Pathogens	Dose	Reference
*Lychas mucronatus *	Mucroporin	*S. subtilis *	50 *μ*g/mL	
		*S. aureus *	25 *μ*g/mL	[39]
	Mucroporin-M1	*S. subtlis *	25 *μ*g/mL	
		*S. aureus *	5 *μ*g/mL	

*Mesobuthus martensii Karsch*	Bmkn2	*S. aureus *	6.25 *μ*g/mL	
		*E. coli *	>100 *μ*g/mL	
	Kn2-7	*S. aureus *	6.25 *μ*g/mL	[40]
		*E. coli *	>100 *μ*g/mL	

*Mesobuthus eupeus *	Meucin-24, 25	*P. berghei *	—	[41]
	Meucin-18	*B. megaterium *	0.25 *μ*M (#)	
		*S. typhimurium *	10.9 *μ*M (#)	[42]
	Meucin-13	*B. megaterium *	0.25 *μ*M (#)	
		*S. typhimurium *	>50 *μ*M (#)	

*Buthus martensii Karsch*	Bmkb1	*S. aureus *	16 *μ*g/mL	
		*E. coli *	18.1 *μ*g/mL	[43]
	Bmkn2	*S. aureus *	0.6 *μ*g/mL	
		*E. coli *	1.5 *μ*g/mL	

*Chaerilus tricostatus *	Ctriporin	*B. subtilis *	10 *μ*g/mL	
		*S. aureus *	5 *μ*g/mL	[44]
		*M. luteus *	5 *μ*g/mL	

*Parabuthus schlechteri *	Parabutoporin	*K. pneumoniae *	1.6 *μ*M	[45]
		*E. coli *	3.1 *μ*M	

*Isometrus maculatus *	Imcroporin	*M. luteus *	20 *μ*g/mL	
		*S. aureus *	20 *μ*g/mL	[46]
		*B. subtilis *	50 *μ*g/mL	

*Opisthacanthus madagascariensis *	IsCT	*S. aureus *	0.7 *μ*M	
		*E. coli *	3.3 *μ*M	[47, 48]
	IsCT2	*S. aureus *	0.7 *μ*M	
		*E. coli *	3.4 *μ*M	
	[A6]-IsCT	*S. aureus *	>64 *μ*M	
		*E. coli *	64 *μ*M	
	[L6]-IsCT	*S. aureus *	64 *μ*M	
		*E. coli *	16 *μ*M	
	[K7]-IsCT	*S. aureus *	1 *μ*M	[49]
		*E. coli *	2 *μ*M	
	[L6,K11]-IsCT	*S. aureus *	2 *μ*M	
		*E. coli *	2 *μ*M	
	[K7,P8,K11]-IsCT	*S. aureus *	2 *μ*M	
		*E. coli *	2 *μ*M	

*Opisthophtalmus carinatus *	Opistoporin-1	*Gram *−	1.3–25 *μ*M	
		*Gram + *	>50 *μ*M	[45]
	Opistorporin-2	—	—	

*Androctonus australis *	Androctonin	*M. luteus *	0.5–1.5 *μ*M	[50, 51]
		*A. viridans *	0.25–0.6 *μ*M	
		*P. syringae *	1.5–3 *μ*M	[50]
		*S. typhimurium *	3–6 *μ*M	

*Pandinus imperator *	Pandinin-1	*Gram + *	1.3–5.2 *μ*M	
		*Gram *−	>20.8 *μ*M	[52]
	Pandinin-2	*Gram + *	2.4–4.8 *μ*M	
		*Gram *−	19.1–38.2 *μ*M	
		*B. subtilis *	1 *μ*M	[53]
	Scorpine	*K. pneumoniae *	10 *μ*M	
		*P. berghei *	0.7 *μ*M*	

*Hadrurus aztecus *	Hadrurin	*Gram + *	<10 *μ*M	[52]
		*Gram − *	>40 *μ*M	

*Hadrurus gertschi *	HgeScplp1	*B. subtilis *	0.2–0.5 *μ*M (#)	
		*S. aureus *	0.2–0.5 *μ*M (#)	
	Hge*β*KTx	*B. subtilis *	0.2–0.5 *μ*M (#)	[54]
		*S. aureus *	>0.5 *μ*M (#)	
	Hge36	*B. subtilis *	>0.5 *μ*M (#)	
		*S. aureus *	>0.5 *μ*M (#)	

*Centruroides noxius *	Noxiustoxin	*P. berghei *	0.7 *μ*M (+)	[55]

*Scorpiops tibetanus *	StCT1	*S. aureus *	12.5 *μ*g/mL	[56]
		*M. luteus *	100 *μ*g/mL	
	StCT2	*S. aureus *	6.25 *μ*g/mL	[56]

*Vaejovis mexicanus smithi *	VmCT1	—	5–25 *μ*M	[57]
	VmCT2	—	10–20 *μ*M	

*Heterometrus laoticus *	HS-1	*K. pneumonia *	—	
		*B. subtilis *	—	[58]
		*P. aeruginosa *	—	

*Heterometrus spinifer *	HsAP2, 3, 4	—	—	[59]

*Heterometrus xanthopus *	Whole venom	—	—	[60]

The dose represents the MIC, the EC_50%_ (+), or the EC_100%_ (#) of the polypeptide, or LD_100%_ (lethal dose ∗) of the polypeptide.

**Table 3:** List of chosen antiviral peptides originating from scorpions.

scorpion	Fraction	Viruses	Dose	Reference
*Lychas mucronatus *	Mucroporin	HIV-1	—	[70–73]
		HBV		
	Mucroporin-M1	HIV-1	—	
		HBV	87 *μ*M (+)	
	Mucroporin-S1	HIV-1	>100 *μ*g/mL	
*Mesobuthus martensii Karsch*	Bmkn2	HIV-1	—	
	Kn2-7	HIV-1	2.76 *μ*g/mL (+)	
*Chaerilus tricotanus *	Ctriporin	HBV	—	
*Heterometrus petersii kills *	Hp1090	HCV	5 *μ*M (+)	
	Mucroporin-M1	Measles	3.52 *μ*M (+)	
		SARS-CoV	7.12 *μ*M (+)	
		H5N1	1.03 *μ*M (+)	

The dose represents the MIC, the EC_50%_ (+), or the EC_100%_ (#) of the polypeptide.

**Table 4:** Summary of the modus operandi of some scorpion derivatives with potential therapeutic use.

Peptides	Origin	Biological effects
Immune diseases		
Charybdotoxin [84, 85]	*L. quinquestriatus *	Blockade of shaker potassium channels (Kv1.3.) which suppresses lymphocytes proliferation and IL2 production.
Parabutoporin [26, 45, 95, 96]	*P. schlechteri *	Activation of the Rac receptor coupled to G protein, and inhibiting NADPH/oxidase complex function. The cumulus of these actions enhances exocytosis and chemotaxis and prevents superoxide production.
Fraction 1 [88]	*A. australis *	Stimulating lymphocytes proliferation.
Total venom [82, 86] *γ*TsTx (gamma toxin) [89]	*T. serrulatus *	Lymphocytes proliferation enhancement. Lymphocytes mobilization from the bone marrow. Pro- and anti-inflammatory factor release (exocytosis). Platelet-activating factor receptor (PAFR), chemotaxis, and complement stimulation.

Cardiovascular diseases		
*γ*TsTx (gamma toxin)[89]	*T. serrulatus *	PAFR stimulation (aggregating factor)
Charybdotoxin [84]	*L. quinquestriatus *	Blockade of voltage and Ca^2+^-activated potassium channels, decreasing prothrombinase activity and phosphatidylserine exposition (aggregating factor).
SVAP [74, 77]	*B. m. Kirsch *	Increasing PGI2 production/altering pro- and anti inflammatory compounds release, probably from white blood cells.
Bpps (Bppk12) [10]	*B. occitanus *	Inhibiting the downregulation of the bradykinin (vasodilatator) and the angiotensin II (vasoconstrictor) synthesis.
Hypotensins [87]	*T. serrulatus *	Inhibiting the downregulation of the bradykinin and enhancing NO release.

Neurological diseases		
Various [102]	*Various *	Interaction with adrenergic and cholinergic receptors.
K+ channels blockers [103]	*Various *	Blockade of potassium current which prevents symptoms and signs in multiple sclerosis.
Whole venom [104] Bmk AS [105, 106]	*B. m. Kirsch *	Inhibition of the voltage-sensitive sodium channels.

Cancer		
Chlorotoxin [107–110] rBmKTa (recombinant) [111]	*L. q. hebraeus* *B. m. Kirsch *	Carrying gene transfecting-nanovecteors to their targeted cancer cells. blockade of chloride channels responsible for cell motility and metastasis invasion.
AmmTx3 [112] BmTx3[112] BeKm-1[112]	*A.m. mauritanicus* *Meso. martensi* *Meso. eupus *	Blockade of hERGK+ channels involved in tumoral cells activities.
BmHYA1 [30]	*B. martensi *	Modulation of cell cycle, apoptosis and invasion. Facilitation of drugs biodistribution by acting on MPPs. Modulation of CD44 surface marker of breast cancer cells.
PESV [113]	*Nd *	Enhances the immune sentinel against tumors.

Osteoporosis		
Whole venom [114]	*H. bengalensis*	Stimulation of osteoclast activity and mineral deposits and modulation the release of osteoporosis regulating factors (antiosteoporosis).
KTXs [115]	*Various *	Blockade of Kv1.3 channels which reduce inflammatory bone resorption.

## List of Abbreviations

(*α*, *β*)-NaTxs: Scorpion toxins affecting *α* or *β*-sodium channels activities
(*α*, *β*, *γ*)-KTxs:*α*, *β*,: Scorpion toxins affecting or *γ*-potassium channels activities
[Ax, Lx, Kx, Px]-IsCT: Modified IsCT by substituting the aminoacids (A, L, K, P) at the corresponding position (x)
Akt: Serine/threonine protein kinase
Amm VIII: *Androctonus mauretanicus mauretanicus* toxin VIII
AmmTx3: *Androctonus mauretanicus mauretanicus* toxin 3
AMP: Antimicrobial peptides
ATP: Adenosine triphosphate
Bekm-1: *Mesobuthus eupeus* toxin 1
BmAP1: *Buthus martensii* Karschactive peptide 1
BmHYA1: *Buthus martensii* hyaluronidase 1
BmkAS: *Buthus martensii *Karsch active substance
Bmkb1: *Buthus martensii *Karsch toxin b1
Bmkn2: *Mesobuthus martensii *Karsch toxin n2
BmkTa: *Buthus martensii *Karsch toxin a
BmTx3: *Mesobuthus martensii* toxin 3
BPP: Bradykinin potentiating peptide
Bppk-12: *Buthus occitanus* bradykinin potentiating peptide K-12
CCR5: Chemokines receptor type 5
CFTR: Cystic fibrosis transmembrane conductance regulator chloride channels
CXCR4: Chemokines receptor type 4
DNA: Deoxyribonucleic acid
DVAP: Scorpion venom active peptide
FDA: Food and drug administration, USA
gp120: Globular protein 120
HBV and HCV: Hepatitis viruses type B and C
hERG: Human ether-a-go-go-related potassium channels family (or KNCH)
Hge36: *Hadrurus gertschi* toxin with 36 amino acids
HgeScplp1: *Hadrurus gertschi* scorpion plp1
Hge*β*KTx: *Hadrurus gertschi* toxin affecting *β*-potassium channels
HIV: human immunodeficiency virus type 1
Hp1090: *Heterometrus petersii kills* fraction 1090
HS-1: *Heterometrus laoticus* scorpine-1
HsAP (x): *Heterometrus spinifer* active peptide (x)
IL-10: Interleukin 10
IL-2: Interleukin 2
IsCT(x): *Opisthacanthus madagascariensis* cytotoxins
KCa (x·y): Calcium-dependent potassium channels subfamily
Kir4.2: Inward current-potassium channels rectifier family 4.2
Kn2-7: *Mesobuthus martensii Karsch* toxin n2-7
Kv (x·y): Voltage-sensitive potassium channels subfamily
LqqIT2: *Leiurus quinquestriatus quinquestriatus* insect toxin 2
Nav (x·y): Voltage-sensitive potassium channel subfamily
NADPH: Nicotinamide adenine dinucleotide phosphate
NO: Nitric oxide
P21: Tumor suppressor gene p21
PAF: Platelet activating factor
PESV: Peptide extracted from scorpion venom
PKC: Phosphokinase C
PLAs2: Phospholipase type 2 (II)
Rac: Receptor-activating chemotaxis
RNA: Ribonucleic acid
ShK (L5): *Stichodactyla helianthus* potassium-channels inhibitor peptide
StCT(x): *Scorpiops tibetanus* cytotoxins (x)
TGF-beta1: Tumor growth factor *β*1
VEGF: Vascular endothelial growth factor
VmCT(x): *Vejovis mexicanus smithi* cytotoxin (x)
*γ*-TS: *γ*-*Tityus serrulatus* toxin.
